# Multivariate Analysis of Morpho-Physiological Traits Reveals Differential Drought Tolerance Potential of Bread Wheat Genotypes at the Seedling Stage

**DOI:** 10.3390/plants10050879

**Published:** 2021-04-27

**Authors:** Mohammed Mohi-Ud-Din, Md. Alamgir Hossain, Md. Motiar Rohman, Md. Nesar Uddin, Md. Sabibul Haque, Jalal Uddin Ahmed, Akbar Hossain, Mohamed M. Hassan, Mohammad Golam Mostofa

**Affiliations:** 1 Department of Crop Botany, Bangladesh Agricultural University, Mymensingh 2202, Bangladesh; mmu074@bsmrau.edu.bd (M.M.-U.-D.); nesar.uddin@bau.edu.bd (M.N.U.); mshaqcb@bau.edu.bd (M.S.H.); 2 Department of Crop Botany, Bangabandhu Sheikh Mujibur Rahman Agricultural University, Gazipur 1706, Bangladesh; jahmed06@bsmrau.edu.bd; 3 Plant Breeding Division, Bangladesh Agricultural Research Institute, Gazipur 1701, Bangladesh; motiar_1@yahoo.com; 4 Bangladesh Wheat and Maize Research Institute, Dinajpur 5200, Bangladesh; akbarhossainwrc@gmail.com; 5 Department of Biology, College of Science, Taif University, P.O. Box 11099, Taif 21944, Saudi Arabia; m.khyate@tu.edu.sa; 6 Department of Biochemistry and Molecular Biology, Bangabandhu Sheikh Mujibur Rahman Agricultural University, Gazipur 1706, Bangladesh

**Keywords:** wheat, drought, robust hierarchical co-cluster, principal component analysis, linear discriminant analysis

## Abstract

Drought is one of the foremost environmental stresses that can severely limit crop growth and productivity by disrupting various physiological processes. In this study, the drought tolerance potential of 127 diverse bread wheat genotypes was evaluated by imposing polyethylene glycol (PEG)-induced drought followed by multivariate analysis of several growth-related attributes. Results showed significant variations in the mean values of different morpho-physiological traits due to PEG-induced drought effects. Correlation analysis revealed that most of the studied traits were significantly correlated among them. The robust hierarchical co-clustering indicated that all the genotypes were clustered into four major groups, with cluster 4 (26 genotypes) being, in general, drought-tolerant followed by cluster 1 (19 genotypes) whereas, cluster 2 (55 genotypes) and 3 (27 genotypes) being drought-sensitive. Linear discriminant analysis (LDA) confirmed that around 90% of the genotypes were correctly assigned to clusters. Squared distance (*D^2^*) analysis indicated that the clusters differed significantly from each other. Principal component analysis (PCA) and genotype by trait biplot analysis showed that the first three components accounted for 71.6% of the total variation, with principal component (PC) 1 accounting for 35.4%, PC2 for 24.6% and PC3 for 11.6% of the total variation. Both PCA and LDA revealed that dry weights, tissue water content, cell membrane stability, leaf relative water content, root-shoot weight ratio and seedling vigor index played the most important discriminatory roles in explaining drought tolerance variations among 127 wheat genotypes. Our results conclude that the drought-tolerant and -sensitive wheat genotypes identified in this study would offer valuable genetic tools for further improvement of wheat productivity in arid and semi-arid regions during this time of unpredictable climate change.

## 1. Introduction

The bread wheat (*Triticum aestivum* L.) is an important cereal crop cultivated across diverse environments, ranging from warm lowlands to temperate highlands [1]. Among the cereals, wheat ranks first and second globally in terms of acreage (215.9 million ha) and production (765.7 million tons), respectively [2], while second in Bangladesh in terms of acreage (0.33 million ha) and production (1.02 million tons) in 2018–2019 wheat growing season [2,3]. In Bangladesh, wheat is cultivated under non-irrigated conditions during the dry winter (November to April) season [4]. The north-western region, namely, the Barind tract, is the major wheat-growing region and one of the largest drought-affected areas of Bangladesh [5].

Drought is one of the major abiotic stresses constraining crop productivity worldwide, it reduces plant productivity by inhibiting growth and development [6]. Drought severely limits wheat productivity and in dry environments, wheat production can be depressed by 50–90% of the crop potential [7]. Moreover, the recent global warming phenomenon is giving rise to an aggravating climatic instability that adversely affects ecosystem quality, plant growth, and agricultural production [8,9]. Global warming and the forcing factors of climate change suggest that more frequent, longer and severe droughts are expected in the 21st century across many regions of the world [10,11,12]. Projected climate changes for Bangladesh include more erratic rainfall resulting in increasing droughts, especially in drier northern and western regions of the country [13]. This predicted drought severity will constrain wheat cultivation and productivity in the future due to the lack of drought-tolerant varieties since the modern wheat varieties are not sufficiently tolerant against abiotic stresses [14]. Therefore, the effort ought to be made to minimize the yield reduction by screening or developing drought-tolerant wheat varieties.

It becomes crucial to screen drought-tolerant wheat genotypes under actual dry environmental condition [15] as drought cannot be easily maintained in the field because of different precipitations that can hamper water deficit [16]. Seed germination and early seedling growth are potentially the most critical stages for water stress [17]. Thus, in vitro screening method is evidently effective in the selection of drought-tolerant wheat genotypes. Many chemical desiccants can be used for inducing in vitro drought stress. Polyethylene glycol (PEG) acts as osmoticum to reduce water potential of culture medium, thus creating drought stress on plant tissues by the outward flow of water from plant tissues to a concentrated solution of PEG [18]. PEG molecules are inert in nature, non-ionic, and induce uniform drought stress without entering the plant cells [19]. Many early drought screening studies had also involved PEG-6000 solutions for induction of dehydration or drought under controlled environments [20,21,22,23,24,25,26,27]. However, in the context of this paper, we will use the terminology ‘PEG-induced drought’.

PEG-6000 induced drought can alter many morphological, phenological, and physiological characters of wheat seedlings. Seedling’s shoot and root length and biomasses were reduced with a decrease in osmotic potential [25]. Other researchers [27,28,29] also assessed the decline in the growth, length, and weight of seedlings in PEG-induced drought conditions. The superior seedling dry weight under PEG stress has been considered as a reliable drought-tolerant criterion for different plant species, including wheat [30]. Many researchers suggested root-to-shoot ratio [31] and seedling vigor [32] could be used as selection criteria for drought tolerance in wheat. Relative water content (RWC) and cell membrane stability (CMS) are useful indices for the rapid evaluation of drought response in wheat breeding. RWC is a good indicator for the selection of drought-tolerant wheat genotypes at the seedling stage [29,33,34]. RWC in terms of its relationship with the volume of cell can correctly show the balance between water absorbed by the plant and released through transpiration [35]. CMS is used as a selection criterion for drought and heat stress tolerance at the seedling stage by many researchers [29,34,36]. The above traits, singly or collectively, are considered an important selection tool for improvement against drought stress due to their relationship with the adaptation mechanisms of plants under stressful conditions [37].

Nonetheless, drought tolerance is not often discussed as an independent character by plant breeders because tolerance mechanisms can be fairly general and polygenic in nature [38]. However, multivariate analysis techniques can be used to explore relationships, classification and parameter prediction within complex data sets as the conclusions are more realistic, meaningful and accurate [39]. Among the multivariate techniques, the robust hierarchical co-cluster (RHCOC) approach produces a far lower clustering error rate than the conventional hierarchical clustering approaches in presence of outlying observations in the dataset [40]. PCA-biplot is one of the most effective multivariate analyses to evaluate the traits interaction and genotypic performance and extensively used to dissect the traits correlation in different crop plants [41]. Linear discriminant analysis (LDA), particularly useful in defining groups of the genotypes as prior classification criteria, identify misclassification error and measure the distance between groups, is effectively used for screening of flooding tolerant mungbean genotypes [42].

Therefore, the overall aim of this exploratory study is to evaluate a large number of wheat genotypes for drought tolerance potential based on the performance of seedling traits under PEG induced drought stress. Specific objectives are framed to-(i) determine the changes in morpho-physiological traits of the wheat genotypes exposed to PEG induced drought stress; (ii) classify wheat genotypes into different clusters using robust hierarchical co-cluster algorithm; and (iii) establish and verify the association between seedling traits and drought tolerance using different multivariate analysis tools.

## 2. Materials and Methods

### 2.1. Plant Materials and Stress Treatment

One hundred twenty-seven diverse wheat genotypes collected from different sources were used in this exploratory study. Among the genotypes, 14 mutant lines (material: BARI Gom 25; mutagen: 1% EMS) were collected from ACI Seed; 17 variety and 1 advanced line from Bangladesh Wheat and Maize Research Institute and Bangladesh Institute of Nuclear Agriculture; and 95 wheat accessions collected from Plant Genetic Resource Center of Bangladesh Agricultural Research Institute. The experiment was laid out in a two factor completely randomized design (CRD) with 3 replicates. The factors included 127 wheat genotypes and two levels of drought stresses simulated by adding polyethylene glycol (PEG)-6000 at two concentrations—0% (control), and 25% (*w*/*v*) having water potential, Ψ_w_ ≈ −0.99 MPa; [43]). PEG is of a high molecular weight and cannot pass through the cell wall, therefore PEG is used to regulate the water potential in simulating the drought stress.

Uniform sized seeds of wheat genotypes were selected and surface-sterilized with 1% sodium hypochlorite for 10 min followed by washing several times with sterile distilled water. The seeds were then soaked in distilled water for 10 min and 30 seeds were sown in two sets of petriplates (11 cm diameter) filled with sterile sand moistened with distilled water for germination for five days in room temperature (28–33 °C). At the 6th day, seedlings were moved to a controlled environment chamber (Model: GC-560H, Firstek Scientific, Taiwan) maintaining 25 ± 1 °C temperature during day and night, relative humidity (RH) of 75–80%, 14 h of photoperiod with photosynthetic photon flux density (PPFD) of 200 μmol m^−2^ s^−1^ using cool-white florescent lamps. At the 9th day of seed sowing, the germination percent was calculated as n/N × 100 (where n was the number of total germinated seed; N was the number of total seeds) (Table S1) and the seedlings were then thinned to 15 in each petriplate. In one set of petriplate, 25% PEG-6000 solution was applied on days 11 and 13 (5 mL petriplate^−1^) of seeds sowing to induce drought stress. Only distilled water was applied to the other set of petri plates and considered as control. After PEG treatment is started, 7 mL of half-strength Hoagland’s nutrient solution was added in petriplates every second day. Seedlings were then allowed to grow up to the 20th day of seed sowing to observe the visual effects of PEG induced drought stress. An example of the experimental setup and the differential effects of PEG-induced drought was illustrated in the Figure S1. Growth chamber temperature and RH were monitored by a digital humidity and temperature meter (Model: HD-306, HTC Instruments, Taiwan). Sources and types of 127 genotypes listed in Table S1.

### 2.2. Measurement of Seedling Traits

In Day 20 after seeds sowing and 9 days since first application of PEG, shoot and root length, fresh and dry weight was recorded from 10 seedlings. Shoot and root length was measured from the root-shoot junction to the tip of the longest leaf and root, respectively. Shoot and root dry weight was recorded after oven drying at 80 °C for 24 h. The root-shoot ratio was calculated as the root dry weight divided by the shoot dry weight. Tissue water content (TWC) in terms of the amount of water per unit shoot or root fresh weight, was calculated following the formula cited from Mickky [44].

TWC = (Fresh wt. − Dry wt.)/(Fresh wt.)

Seedling vigor index (SVI) was calculated according to the formula proposed by Hellal et al. [22] as

SVI = ((Shoot length + Root length) × Germination percent)/100

Leaf relative water content (LRWC) was estimated according to the procedure of Meher et al. [23]. Briefly, about 0.5 g of leaf sample was incubated in 100 mL of distilled water for 4 h. After that, the turgid weights of leaf samples were taken. The leaf samples were oven-dried at 80 °C for 24 h. The dry weights of the samples were taken till a constant weight was achieved.

LRWC (%) = [(Fresh wt. − Dry wt.)/(Turgid wt. − Dry wt.)] × 100

Cell membrane stability (CMS) was determined following the procedure of Sairam et al. [45]. Briefly, leaf samples (0.1 g) were cut into uniformly sized squares and placed in test tubes containing 10 mL of deionized water in two sets. One set was kept at 40 °C for 30 min and another set at 100 °C in a boiling water bath for 15 min and their electric conductivities C_1_ and C_2_, respectively, were measured by a conductivity meter (Model: EC-400L, HumanLab Instrument Co., Suwon, Korea).

CMS (%) = [1 − (C_1_/C_2_)] × 100

Expanded leaves from 10 seedlings were collected to form a single replicate and the same repeated twice from an independent set of seedlings to obtain three biological replicates. All the measurements and assays were done in triplicate.

The stress tolerance index (STI) for all traits was calculated using the following formula used by Fernandez [46].

$$\mathrm{STI}=(X_{c}\times X_{s})/{\left({\bar{X}}_{c}\right)}^{2}$$

where X_c_ and X_s_ indicate the observed values of a trait in a given genotype under control and stress treatments, respectively, while ${\bar{X}}_{c}$ is the average value of a particular trait examined in all genotypes under non-stress condition.

### 2.3. Statistical Procedure and Computer Analysis of Data Using Machine Learning Algorithms

All the statistical analyses were done using R-4.0.2 for win (http://CRAN.R-project.org/) (accessed on 23 February 2021) in Rstudio-1.3.1093 (https://rstudio.com/) (accessed on 23 February 2021). Data obtained were subjected to 2-factor (stress treatment × genotypes) analysis of variance (ANOVA) in the general linear model using the package lme4 [47] and the mean differences were compared by Tukey’s HSD test using the library agricolae [48]. Differences at *p* < 0.05 were considered significant.

For the graphical presentation of the descriptive statistics of the traits, box and whisker plots were used. The relative magnitude of change in the trait values due to PEG-induced drought was displayed by radar plot. Boxplot and radar plots were prepared using the packages ggplot2 and fmsb, respectively, along with reshape2 [49,50].

The degree of association of the studied traits was determined by correlation coefficients among them. The correlation coefficient matrix and correlation heatmap were visualized using ggpair function of the packages GGally and ggplot2 [51].

To categorize the similar genotypes in clusters, the hierarchical co-cluster algorithm was used. The extracted clusters were distinct from each other, whereas the genotypes within each cluster were broadly similar to each other. The STIs of the studied traits were normalized and adapted to the library rhcoclust to generate robust hierarchical co-clusters and cluster heatmap [40]. Prior to cluster analysis, the number of clusters was determined using gap statistic algorithm in fviz_nbclust function of factoextra.

The principal component analysis (PCA) was used to reduce the dimensionality of the dataset without losing important information. The Eigen value, latent vectors and PCA-biplot extracted from the PCA. The PCA was carried out using the packages ggplot2, factoextra and FactoMineR [50,52].

Linear discriminant analysis (LDA) was used to determine the correctness of the prior classification of the genotypes by cluster analysis and to calculate the squared distances among the clusters. LDA was performed by using the packages MASS, tidyverse and caret [53].

## 3. Results

### 3.1. Mean Variability in Seedling Traits

**Figure 1:**
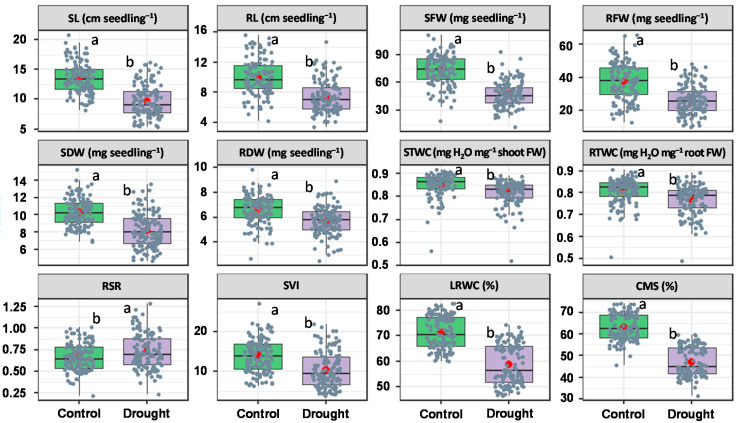
Box plots showing the descriptive statistics of the seedling traits measured from 127 wheat genotypes under control and PEG-induced drought stress. Different letter(s) on the boxes indicate a significant difference at *p* < 0.05 by Tukey’s HSD. The horizontal line and red circle within the box represent the median and mean, respectively. The lower and upper limit of the box, lower and upper whisker represents Q1 (first quartile/25th percentile), Q3 (third quartile/75th percentile), (Q1−1.5IQR) and (Q3 + 1.5IQR), respectively. IQR—interquartile range. Slate color dots on the boxes indicate the distribution of 127 observations. (SL—shoot length; RL—root length; SFW—shoot fresh weight; RFW—root fresh weight; SDW—shoot dry weight; RDW—root dry weight; STWC—shoot tissue water content; RTWC—root tissue water content; RSR—root-shoot weight ratio; SVI—seedling vigor index; LRWC—leaf relative water content; CMS—cell membrane stability).

In the experimental setup including 127 wheat genotypes and two growing conditions, highly significant variation was unveiled among the wheat genotypes in both growing conditions for all seedling traits (Table S2). The descriptive statistics of the seedling traits were presented in the box plot (Figure 1). The trait values were decreased significantly in PEG treated seedlings, except the root-shoot ratio (RSR), which showed a significant increase under PEG induced drought.

PEG treated seedlings showed a substantial decrease in shoot and root traits. Due to PEG treatment, a comparatively higher decrease in shoot traits were recorded than the root traits (Figure 1). Shoot length (SL), shoot fresh weight (SFW), shoot dry weight (SDW) and shoot tissue water content (STWC) were decreased by 29%, 36%, 20% and 4%, respectively, over control, whereas 26%, 29%, 14% and 5% decrease was recorded in root length (RL), root fresh weight (RFW), root dry weight (RDW) and root tissue water content (RTWC), respectively, by PEG induced drought treatment (Figure 1).

Contrarily, a significant increase in root-shoot ratio (RSR) was observed in drought-stressed seedlings (Figure 1). As a result of drought stress, 10% increase in the RSR was recorded while 25% increase was observed in the seedling vigor index (SVI) (Figure 1). Leaf relative water content (LRWC) and cell membrane stability (CMS) were decreased by 17% and 26%, respectively, due to PEG induced drought (Figure 1).

### 3.2. Correlation Analysis

**Figure 2:**
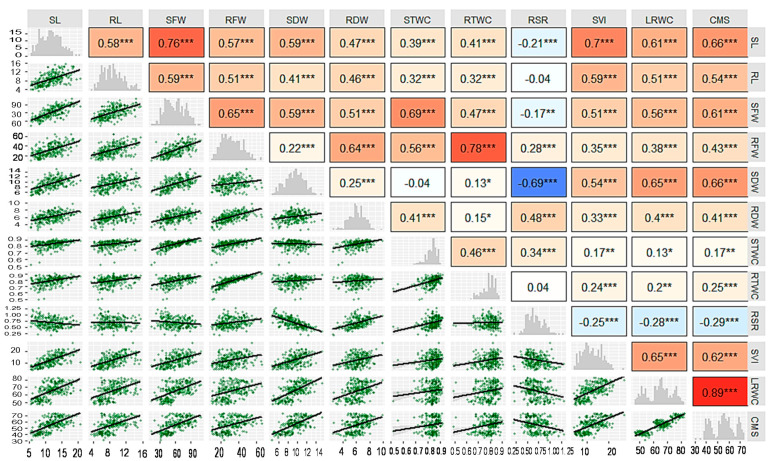
Scatterplot, correlation matrix and heatmap of the studied seedling traits of 127 wheat genotypes grown under control and PEG-induced drought stress. In the upper panel, red and blue boxes indicate positive and negative correlations, respectively, with increasing color intensity reflects a higher coefficient. The diagonal panel indicates the distribution histogram of correlated traits. The lower panel indicates a scatterplot and trendline of the correlated traits. *, ** and *** indicate significant at *p* < 0.05, *p* < 0.01 and *p* < 0.001. (SL—Shoot length (cm seedling^−1^); RL—root length (cm seedling^−1^); SFW—shoot fresh weight (mg seedling^−1^); RFW—root fresh weight (mg seedling^−1^); SDW—shoot dry weight (mg seedling^−1^); RDW—root dry weight (mg seedling^−1^); STWC—shoot tissue water content (mg H_2_O mg^−1^ shoot FW); RTWC—root tissue water content (mg H_2_O mg^−1^ root FW); RSR—root-shoot weight ratio; SVI—seedling vigor index; LRWC—leaf relative water content (%); CMS—cell membrane stability (%)).

The degree of association among the traits was determined by their correlation coefficients. Pearson correlation analysis revealed a significant relationship among observed seedling traits (Figure 2). All shoot and root traits were significantly correlated among them, except the correlation between SDW and STWC. Apart from the root traits, RSR showed a significant negative correlation with all other seedling traits, except with STWC. It was observed that RSR maintained a positive and significant correlation with RFW and RDW whereas, the correlation with RL (negative) and RTWC (positive) were non-significant.

### 3.3. Hierarchical Clustering and Co-Clustering of Genotypes and Traits

**Figure 3:**
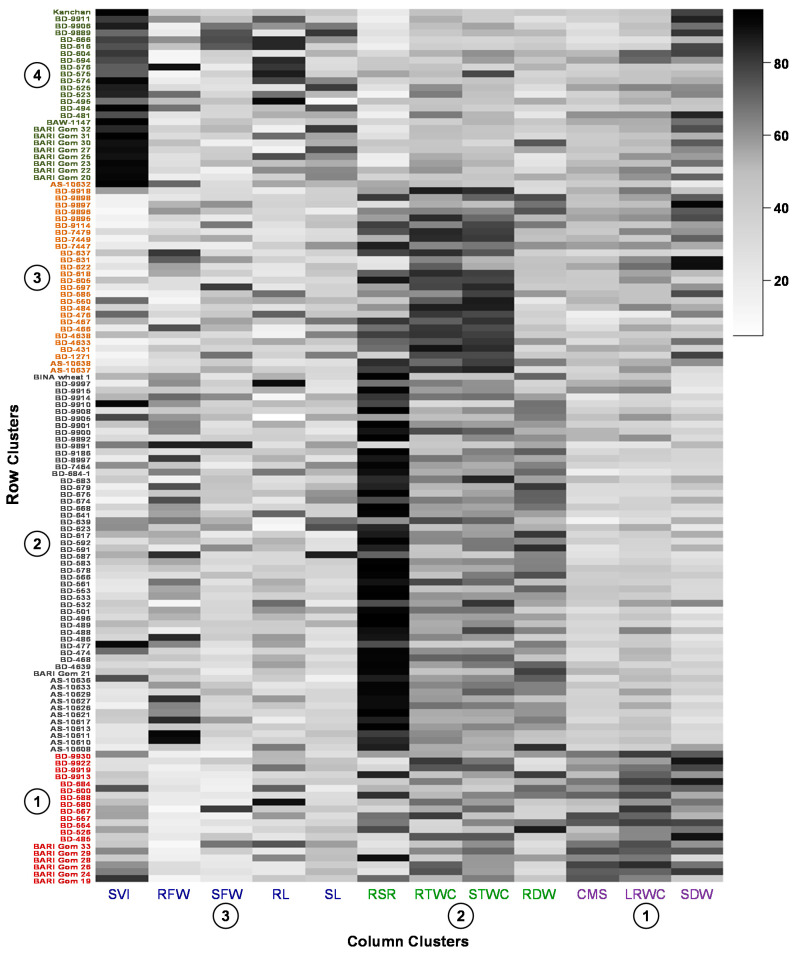
Robust hierarchical co-clustering (method = ward. D2 and distance = Manhattan) indicates trait and genotypes association. The STI (stress tolerance index) values obtained from studied traits of the wheat genotypes were normalized and clustered. Four-row clusters (cluster numbers were determined by the machine language of gap statistic) were obtained at the genotype level (row cluster-1, 2, 3 and 4) and three-column clusters (column cluster-1, 2 and 3) were obtained at the trait level. Different greyscale shades express the intensity of the transformed STI values of the traits. List of the genotypes in each cluster is presented in the Table S3. (SL—shoot length; RL—root length; SFW—shoot fresh weight; RFW—root fresh weight; SDW—shoot dry weight; RDW—root dry weight; STWC—shoot tissue water content; RTWC—root tissue water content; RSR—root-shoot weight ratio; SVI—seedling vigor index; LRWC—leaf relative water content; CMS—cell membrane stability).

Prior to the cluster analysis, the number of projected clusters was determined using gap statistic algorithm (Figure S2). Based on the variation in observed traits, 127 genotypes were grouped into four hierarchical row clusters and the traits were grouped into three by using a robust co-clustering algorithm and presented as a co-cluster heatmap (Figure 3; list of the genotypes in each cluster is provided in Table S3). The highly similar genotypes were placed in a row cluster whereas the highly associated traits were placed in a column cluster. The SDW, LRWC and CMS formed column cluster 1. Column cluster 2 contained RDW, STWC, RTWC and RSR and the rest of the traits SL, RL, SFW, RFW and SVI were placed in cluster 3.

Among the row clusters, cluster 2 contained the highest number of wheat genotypes (55) followed by clusters 3 (27), 4 (26) and 1 (19) (Figure 3 and Table 1). In general, row cluster 1 was determined mostly by traits of column cluster 1 and then by cluster 2 traits, while row cluster 2 and 3 dominated by the traits of column cluster 2, though row cluster 4 was governed by the traits of almost all column clusters (Figure 3). Mean stress tolerance index (STI) of SL, RL, SFW, SDW, SVI, LRWC and CMS were the highest in row cluster 4. Apart from these highest contributing traits, other seedling traits also substantially contributed to cluster 4.

The genotypes in row cluster 1 maintained better STI of SDW, SVI, LRWC and CMS followed by cluster 4. The row of cluster 2 was chiefly characterized by the highest STI of RFW, RDW, STWC, RTWC and RSR and well maintained STI of SL, RL and SFW followed by cluster 4. Row cluster 3 had a better preserved STI of STWC, RTWC and RSR followed by row cluster 4 and a decent contribution of SDW.

Except BARI Gom 21 and BINA wheat-1, all the wheat varieties and advanced lines were distributed in the row cluster 1 (6 variety) and 4 (9 variety and 1 line), whereas, most of the mutant lines and wheat accessions were placed in the row cluster 2 and 3. (Figure 3 and Table S3).

### 3.4. Variability of the Genotypes in the Extracted Clusters

**Figure 4:**
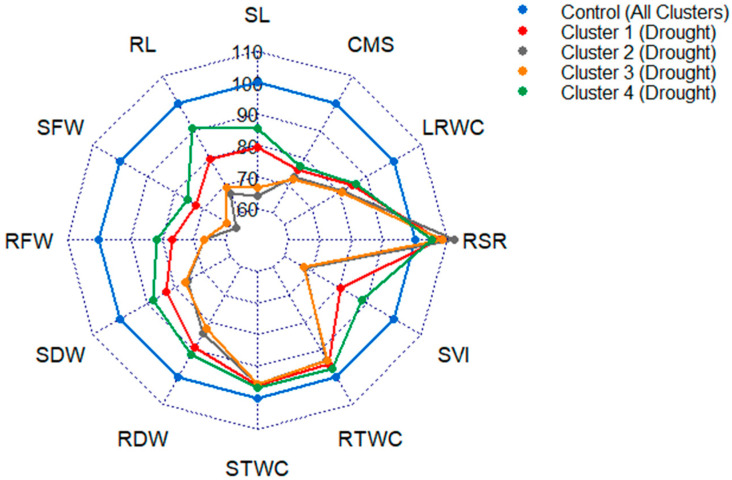
Radar plot showing changes in seedling traits of genotypes of different clusters caused by PEG-induced drought stress. The values are expressed as % of the control. (SL—shoot length; RL—root length; SFW—shoot fresh weight; RFW—root fresh weight; SDW—shoot dry weight; RDW—root dry weight; STWC—shoot tissue water content; RTWC—root tissue water content; RSR—root-shoot weight ratio; SVI—seedling vigor index; LRWC—leaf relative water content; CMS—cell membrane stability).

Results revealed that genotypes of cluster 4 performed significantly better under PEG treatment followed by cluster 1, while cluster 2 and 3 found to be affected substantially as a consequence of PEG-induced drought stress (Figure 4 and Table 2).

Shoot length (SL) found to be decreased by 21%, 36%, 33% and 14% in cluster 1, 2, 3 and 4, respectively, due to drought stress treatment (Figure 4 and Table 2). The percent decrease in RL under PEG treatment compared to control was 21%, 33%, 31% and 9% in cluster 1, 2, 3 and 4, respectively. As a result of drought stress treatment, the least 25% decrease in SFW was recorded in cluster 4 followed by 28% in cluster 1, while on average 41% decrease was recorded in other two clusters, whereas RFW decreased by 23%, 33%, 33%, and 18% in cluster 1, 2, 3 and 4, respectively, compared to control.

Under PEG induced drought treatment, on average 24% decrease in SDW was recorded in cluster 2 and 3, while 17% and 12% decrease were recorded in cluster 1 and 4, respectively, compared to control (Figure 4 and Table 2). A similar pattern of the decrease was observed in RDW with 11%, 16%, 18% and 8% decrease in cluster 1, 2, 3 and 4, respectively, due to drought treatment. Shoot and root tissue water contents were also decreased in a similar trend.

Conversely, RSR was increased under PEG treatment in all clusters, recording an increase by 8%, 12%, 9%, and 5% in cluster 1, 2, 3 and 4, respectively, compared to control (Figure 4 and Table 2). As a result of PEG treatment, the least 12% decrease in SVI was recorded in cluster 4 followed by 20% in cluster 1, while on average 33% decrease was recorded in other two clusters.

Due to PEG induced drought treatment, the least 14% decrease in LRWC was recorded in cluster 4 followed by 15% in cluster 1, while on average 19% decrease was recorded in other two clusters, whereas CMS decreased by 24%, 27%, 28%, and 23% in cluster 1, 2, 3 and 4, respectively, compared to control (Figure 4 and Table 2).

### 3.5. Principal Component Analysis

**Figure 5:**
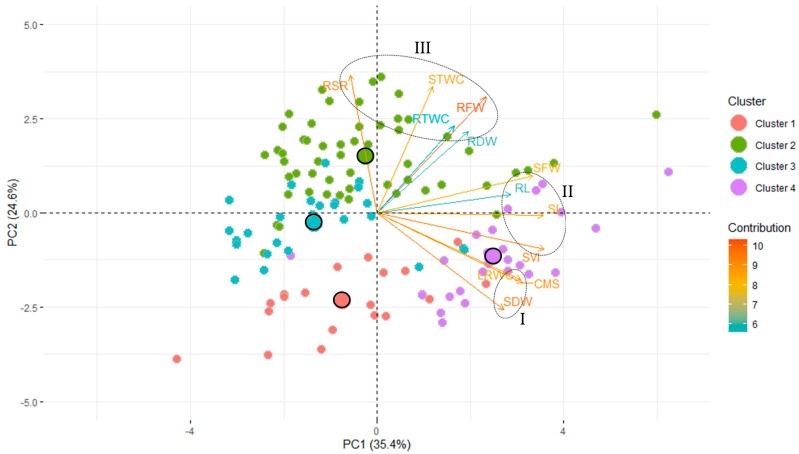
PCA-Biplot of seedling traits and wheat genotypes. Genotypes dispersed in different ordinates based on the dissimilarity among them. The length and color intensity of a vector in the biplot indicate the quality of representation and the contribution of the traits, respectively, on the principal components. The angles between the vectors derived from the middle point of biplots exhibit positive or negative interactions of studied traits. Bigger circles indicate the centroid of the corresponding cluster. (SL—shoot length; RL—root length; SFW—shoot fresh weight; RFW—root fresh weight; SDW—shoot dry weight; RDW—root dry weight; STWC—shoot tissue water content; RTWC—root tissue water content; RSR—root-shoot weight ratio; SVI—seedling vigor index; LRWC—leaf relative water content; CMS—cell membrane stability).

Principal component analysis (PCA) is a multivariate statistical analysis for examining and simplifying complex and large datasets. Based on the correlation among the traits and extracted clusters, the pattern of variation in wheat genotypes were also studied using principal component analysis (PCA) to evaluate the diversity of the genotypes and their association with the observed traits. The stress tolerance index (STI) of all studied traits were subjected to PCA. A total of 12 principal components (PCs) were obtained, but only three PCs that exhibited eigenvalues > 1 were measured as significant. The rest of the non-significant PCs (eigenvalue < 1) were not worthy of further interpretation. The values of the PCs explained all the characters influencing about 72% of the genotypic variability in PEG stress tolerance that accounted up to the first three components, while the first two PCs explained 60% of the variability (Table 3 and Figure 5).

PCA-biplot showed the PC1 exhibited about 35% of the total variability and explained principally by SVI, SL, SFW, CMS, LRWC, RL, SDW and RFW (Table 3 and Figure 5). The second PC accounted for about 25% of the total variation and are mostly contributed by RSR, STWC, RFW, RTWC and SDW. The PC3 explained about 12% of total variability and are contributed by RDW, RSR and RTWC.

A PCA biplot analysis can be utilized to select traits that can be categorized into main groups and subgroups based on homogeneity and dissimilarity. In our data set, three groups of traits were identified in the PCA biplot considering both PC1 and PC2 simultaneously (Figure 5). The SDW, LRWC and CMS were clustered in group I, while SL, RL, RFW, and SVI were in group II; and RSR, STWC, RTWC, RFW and RDW with group III.

Interestingly, the PCA biplot revealed that group I traits, the major contributors in PC1, were strongly associated with genotypes of row cluster 1 and 4, while the traits of group II, also the contributors in PC1, were associated with the genotypes of row cluster 4 (Figure 5 and Table 3). The traits of group III contributed to PC2 were found to be the most closely correlated with the genotypes of row cluster 2 and 3, however, some traits of group III (RDW, STWC and RTWC) closely linked with the genotypes of row cluster 4 (Figure 5 and Table 3). PCA-biplot also indicated the cluster centroids (the multi-dimensional average of the cluster) and the approximation of distances among them (Figure 5).

### 3.6. Linear Discriminant Analysis

LDA is used to reduce the number of dimensions (i.e., variables) in a dataset while retaining as much information as possible and to redefine groups of the genotypes as prior classification criteria. Seedling traits were ordered by the absolute size of the coefficients with the linear discriminant functions (LD) in Table 4. It was observed that the absolute coefficient for RDW (1.340) was ranked first of the discriminatory variables followed by SDW (0.729), STWC (0.640), CMS (0.538), RTWC (0.518) and LRWC (0.513) indicated the dominant role of the traits in explaining 72% variation under LD1 (Table 4).

On the other hand, the largest absolute coefficients of RSR (1.238) followed by SVI (1.008), SDW (0.837) and RDW (0.589) mostly explained 18% variation in LD2. Variation in LD3 (10%) was dominantly played by RSR (1.631), STWC (1.058) and RDW (0.582). Taken together, the above seedling traits (RDW, SDW, STWC, CMS, RTWC, LRWC, RSR and SVI) played the most dominant discriminatory role in explaining the variation of the 127 wheat genotypes by stepwise linear discriminant analysis.

### 3.7. Verification of Cluster Grouping by LDA

The row clusters of wheat genotypes created using cluster analysis were verified with the predictive ability of linear discriminant analysis (LDA). Genotypes within the prior clusters were tested, compared and assigned in different groups based on LDA and then identified the misclassified genotypes that were re-assigned to the appropriate groups (Table 5). Results of the LDA revealed that about 95%, 86%, 89% and 96% genotypes were correctly assigned to cluster 1, 2, 3 and 4, respectively, with an average of 90% correctness in assigning genotypes to different clusters.

### 3.8. Mahalanobis Distance Matrix

The Mahalanobis squared distance (*D*^2^) among the clusters were calculated by LDA (Table 6). The distance matrix revealed that cluster 2 and 4 were the most distant with 21.02 units followed by distance of cluster 1 and 2 (20.29 units). Cluster 4 assembled the genotypes that performed better in most of the characters under PEG stress followed by cluster 1. In contrast, the genotypes grouped in cluster 2 performed poorly (Table 6). The most similar clusters in the present study were clusters 2 and 3 (distance 7.05 units).

### 3.9. Co-Cluster Based Selection of Genotypes

The robust hierarchical co-clustering is the robust approach for clustering as well as co-clustering row and column entities in the absence and presence of outlying observations in the dataset. The selection of the groups of genotypes depends mainly on the objectives of the breeders in the breeding program. To make the selection process more precise and convenient, we have prepared a co-cluster matrix from 4 row and 3 column clusters extracted from the robust hierarchical co-cluster algorithm (Table 7). Genotypes within the co-clusters were sorted in descending order by the transformed scores using a machine language algorithm adapted for the rhcoclust package.

## 4. Discussion

### 4.1. Trait Variability under PEG-Induced Drought Stress

Due to drought stress, significant changes were observed among the wheat genotypes for all observed traits (Figure 1). Except for RSR, the average values of studied traits were decreased in PEG induced drought-stressed seedlings as compared to the control condition. Similar findings were reported by [54] in wheat plant against drought stress.

Length and fresh weight of shoot and root severely affected under PEG-induced drought stress in the present study (Figure 1). Our results are in agreement with the findings of other researchers who have reported a significant decrease in shoot and root length, shoot and root fresh weight, and tissue water content in wheat [27,28,29,34] and in barley seedling [22] due to PEG-induced drought stress or soil drought. In this study, a higher relative decrease in the above traits of the genotypes of row cluster 2 and 3 suggested their relative sensitivity to PEG induced drought stress than the other clusters (Figure 4 and Table 2).

The dry mass of wheat seedling is an important trait and is also affected by PEG and water-deficient stress. Dry matters accumulation a very strong parameter to realize how much biomass is gained by the seedling. We have recorded a significant but variable extent of decrease in shoot and root dry weight; with the least decrease in genotypes of row cluster 4 followed by cluster 1 (Figure 4 and Table 2). The decreasing trend in seedling dry weight was also reported by other researchers [28,29,34] who found that drought stress had a significant effect on dry matter production of seedlings. The maintenance in seedlings’ dry weight under PEG-induced drought stress has been considered as a reliable drought-tolerant criterion for different plant species, including wheat [30]. A lower relative decrease in shoot and root dry weight of the genotypes of row cluster 4 and 1 indicated that these genotypes were able to endure drought stress better than the genotypes of row cluster 2 and 3 (Figure 4 and Table 2).

The root-shoot ratio (RSR) imitates relative root and shoot growth patterns of a crop plant. In the present study, mean RSR was increased significantly due to drought stress and the increase was comparatively higher in the genotypes of row cluster 2 and 3 than the genotypes of row cluster 1 and 4 (Figure 4 and Table 2). A higher root-shoot ratio indicates the root growth is less affected than shoot growth of the seedlings under PEG treatment. The less affected root growth under drought stress was considered as a drought escaping mechanism of wheat seedlings by other researchers [26,27,55]. Higher RSR of the genotypes of row cluster 2 and 3 in the current study was probably due to a higher reduction in SDW than RDW of the genotypes under PEG-induced drought. In accordance with this belief, the results obtained by Sani and Boureima [56] highlighted that physiological events of the root system are less sensitive to limited water, while the upward movement of the xylem sap is constrained in lower water potential media.

Seedling vigor index (SVI) is an approach to measure stress tolerance of crop at the seedling stage by considering germination percentage, shoot and root lengths together. In our study, the SVI significantly decreased due to PEG-induced drought stress. A lower relative decrease in SVI of row cluster 4 followed by row cluster 1 indicated that the genotypes in these two clusters performed better under PEG stress. Less affected shoot and root length of row cluster 4 and 1 coincided with lesser decrease of SVI (Figure 4 and Table 2). A similar observation was reported by Duman [57] in lettuce, Radhouane [58] in pearl millet, Saha et al. [21] in wheat and Hellal et al. [22] in barley.

Leaf relative water content (LRWC) is a physiological trait that has a great importance on the screening of wheat genotypes for drought tolerance. Drought-induced reduction in the leaf relative water content has been reported in many crops including wheat [29,34,59]. In the present study, LRWC decreased significantly due to PEG stress and the decrease was more prominent in the genotypes of row cluster 2 and 3 than the row cluster 1 and 4 (Figure 4 and Table 2). Relatively higher reduction in LRWC of row cluster 2 and 3 was an indication of the sensitivity of genotypes of those clusters to PEG-induced drought stress. Similar higher reduction in leaf relative water content in drought sensitive wheat genotypes as compared to tolerant ones has been observed earlier [29,60,61].

It is well established that water stress could greatly disturb the stability of plant cellular membranes [62]. Cell membrane stability (CMS) is an efficient physiological criterion while studying drought tolerance. The tolerant genotypes showed a minimal reduction in CMS values under drought stress. Research stated that higher RWC and CMS values were reflecting the higher capability to tolerate the drought stress [63]. The present study demonstrated that CMS decreased significantly under PEG induced drought stress with a relatively lower decrease in the genotypes of row cluster 4 than other row clusters (Figure 4 and Table 2). A similar decrease in the CMS was also reported by Ahmed et al. [29] and Ahmed et al. [34] in wheat seedlings. It was evident that drought-tolerant genotypes maintained greater CMS, which ultimately increased the endurance of genotypes following drought treatments [36].

Taken together, genotypes of different hierarchical row clusters showed differential variation in the performance of studied seedling traits (Figure 4). Due to PEG-induced drought, genotypes of row cluster 4 exhibited a minimal change in the trait performance compared to control followed by cluster 1. Higher relative changes in the traits of row cluster 2 and 3 placed the genotypes to the drought-sensitive end. Those genotypes narrowed down the variation of performance of the studied traits in PEG that could be considered drought tolerant. Placement of wheat varieties in the row cluster 1 and 4 indicated that these varieties may have specific association with better tolerance to PEG-induced drought than most of the mutant lines and accessions (Figure 3 and Table S3). Mutant line AS-10632 and 28 accessions were also distributed to the tolerant clusters.

### 4.2. Association between Traits, Genotypes and Drought Tolerance

Correlation studies illustrate the nature and degree of association between any pairs of parameters. It offers a core concept of the association among various traits, which is beneficial for plant breeders in choosing varieties having desired attributes [64]. With few exceptions, all the studied traits in the present study exhibited significant correlations with each other (Figure 2), which suggesting that change in any one of those traits correspondingly change the other traits. This indicated that these seedling traits of wheat play an important role under PEG induced drought stress conditions to determine the response of drought. It means that if one reliable trait is picked in drought stress and used as a selection criterion that will lead to affect other seedling traits for drought conditions [65].

Hierarchical cluster analysis clearly revealed that genotypes of row cluster 4 have the highest ability to tolerate PEG-induced drought stress as judged by almost all seedling traits (Figure 3 and Table 1). The row of cluster 1 mostly contributed by dry weights and physiological traits could be placed in the second choice for further evaluation. Though row clusters 2 and 3 were less distant, but still have some potentials (STWC, RTWC and RSR) for the breeder’s interest. Cluster analysis has been exploited to define the dissimilarity and grouping of the genotypes based on drought tolerance indices [66,67,68,69]. The successive linear discriminant analysis of the cluster groups exhibited that about 90% of the genotypes were correctly assigned to different clusters that means misclassification of the genotypes was lesser in our used clustering algorithm. We have used a comparatively newer R package rhcoclust for robust hierarchical co-clustering of our data. This algorithm showed far less error rate than other contemporary clustering algorithms when outlying observations present in the dataset [40]. It can also be used to create different trait-genotype clustering matrix that can allow researchers to select genotypes of their desired trait groups. We have also sorted the genotypes in a co-cluster matrix in descending order to select the best performers using this algorithm (Table 7).

PCA is a powerful statistical procedure to reduce the dimensions of the variables and to divulge constructive evidence-driven feedback from a highly correlated dataset [70]. In the PCA biplot, the cosine of the angle between the trait vectors approximates the correlation between them, where an acute angle (<90°) represents positive correlation, angle of >90° indicates a negative correlation, while equivalent to 90° angle denotes traits are independent of each other. It has been previously reported that the angles between the vectors of the traits in biplot analysis do not precisely translate into correlation coefficients [71]. However, our results clearly demonstrated that correlations of a trait pair were well coordinated with the approximation of the vector angles and contribution of the same trait pair in the PCA biplot (Figure 1 and Figure 5). PCA biplot analysis has been used widely and effectively by other researchers for screening drought-tolerant cultivars of wheat [27,34,67,68].

Correlation study, hierarchical cluster analysis and PCA indicated that contrasting variations were present in 127 wheat genotypes due to differences in PEG induced stress tolerance and classified the genotypes into four distinct clusters. LDA then confirms the accuracy of the distribution of the genotypes into different clusters with overall correctness of about 90% (Table 5). Moreover, LDA measured the distance between the hierarchical clusters, an approximation of which was figured out as cluster centroids in the PCA-biplot (Figure 5). LDA also depicted that RDW, SDW, STWC, CMS, RTWC, LRWC, RSR and SVI played the most discriminatory role in the classification of 127 wheat genotypes into four clusters. LDA was effectively used for the screening of flooding tolerant mungbean genotypes earlier [42]. Our results revealed that clustering of the wheat genotypes differing in PEG-induced drought stress tolerance based on the robust hierarchical co-clustering was well interpreted by the results obtained from PCA and LDA. Altogether, it is evident that 127 wheat genotypes exhibited significant variation in the PEG-induced changes in seedling traits and the multivariate analyses could be effective in the identification of the wheat genotypes of desirable traits for drought tolerance.

## 5. Conclusions

Based on seedling traits, some genotypes were found PEG-induced drought stress-tolerant, and some were drought-sensitive. According to the magnitude of change in the seedling traits and the outcome of various multivariate analyses, genotypes of cluster 4 appeared as drought-tolerant trailed by genotypes of cluster 1 because these genotypes performed well in most of the seedling traits studied, whereas genotypes of cluster 2 and 3 were affirmed as sensitive to PEG-induced drought stress due to their poor growth and physiological capability under drought stress. Results of the present study will contribute to understanding the differential responses of bread wheat genotypes to PEG-induced drought stress based on the seedling traits. Furthermore, drought tolerance is not often discussed as an independent character by the plant breeders and thus by using co-cluster combinations, the breeders can effectively choose the genotypes of the trait groups they are interested in.

## Figures and Tables

**Table 1:** Comparison profile of the four clusters of 127 wheat genotypes classified by robust hierarchical clustering (cluster figures are means of STI values for the genotypes in each cluster).

Seedling Traits	Average STI of Clusters			
	Cluster 1	Cluster 2	Cluster 3	Cluster 4
Number of genotypes	19	55	27	26
Shoot length (SL)	0.60	0.69	0.60	1.07
Root length (RL)	0.67	0.78	0.52	1.10
Shoot fresh weight (SFW)	0.44	0.67	0.63	0.87
Root fresh weight (RFW)	0.32	0.99	0.58	0.82
Shoot dry weight (SDW)	0.94	0.63	0.81	1.18
Root dry weight (RDW)	0.71	1.06	0.69	0.85
Shoot tissue water content (STWC)	0.82	1.02	0.96	0.95
Root tissue water content (RTWC)	0.82	0.99	0.96	0.98
Root-shoot weight ratio (RSR)	0.75	1.69	0.83	0.72
Seedling vigor index (SVI)	0.71	0.70	0.43	1.62
Leaf relative water content (LRWC)	0.96	0.77	0.72	1.00
Cell membrane stability (CMS)	0.87	0.69	0.64	0.91

**Table 2:** Mean of measured seedling traits and percent change over control of four-row clusters extracted from 127 wheat genotypes grown under control and drought stress.

Cluster	Treatment	SL	RL	SFW	RFW	SDW	RDW	STWC	RTWC	RSR	SVI	LRWC	CMS
Cluster 1	Control	11.91	9.21	56.50	24.86	10.86	5.76	0.79	0.76	0.54	13.21	74.78	66.93
	Drought	9.44	7.32	40.79	19.10	9.03	5.14	0.76	0.73	0.58	10.60	63.36	50.73
	% change	(−) 20.7 ^b^	(−) 20.5 ^b^	(−) 27.8 ^b^	(−) 23.2 ^b^	(−) 16.9 ^b^	(−) 10.7 ^b^	(−) 3.8 ^ab^	(−) 4.8 ^ab^	(+) 7.7 ^ab^	(−) 19.7 ^b^	(−) 15.3 ^b^	(−) 24.2 ^b^
Cluster 2	Control	13.94	10.66	78.27	44.55	9.27	7.39	0.88	0.83	0.81	13.65	69.17	61.04
	Drought	8.94	7.14	45.22	29.74	7.01	6.22	0.84	0.78	0.91	9.20	56.18	44.71
	% change	(−) 35.9 ^a^	(−) 33.0 ^a^	(−) 42.2 ^a^	(−) 33.2 ^a^	(−) 24.4 ^a^	(−) 15.9 ^a^	(−) 4.4 ^a^	(−) 5.9 ^a^	(+) 12.4 ^a^	(−) 32.6 ^a^	(−) 18.8 ^a^	(−) 26.8 ^a^
Cluster 3	Control	12.64	8.39	72.18	32.97	10.43	5.99	0.85	0.80	0.58	10.59	67.58	59.94
	Drought	8.42	5.81	44.10	21.95	7.98	4.93	0.81	0.76	0.63	7.12	54.56	43.41
	% change	(−) 33.4 ^a^	(−) 30.7 ^a^	(−) 38.9 ^a^	(−) 33.4 ^a^	(−) 23.5 ^a^	(−) 17.7 ^a^	(−) 4.2 ^ab^	(−) 5.8 ^a^	(+) 8.6 ^ab^	(−) 32.8 ^a^	(−) 19.3 ^a^	(−) 27.6 ^a^
Cluster 4	Control	15.03	10.78	77.52	36.12	11.80	6.34	0.84	0.81	0.54	18.86	76.62	68.52
	Drought	12.87	9.78	58.54	29.59	10.36	5.82	0.82	0.79	0.57	16.63	65.82	52.91
	% change	(−) 14.4 ^b^	(−) 9.2 ^c^	(−) 24.5 ^b^	(−) 18.1 ^b^	(−) 12.2 ^b^	(−) 8.2 ^b^	(−) 3.2 ^b^	(−) 2.8 ^b^	(+) 5.3 ^b^	(−) 11.8 ^c^	(−) 14.1 ^b^	(−) 22.8 ^b^

In a column, mean % change values with different superscript letter(s) are significantly different at *p* ≤ 0.05 by Tukey’s HSD. (+) and (−) indicate percent increase and decrease, respectively, due to PEG-induced drought over control. (SL—Shoot length (cm seedling^−1^); RL—root length (cm seedling^−1^); SFW—shoot fresh weight (mg seedling^−1^); RFW—root fresh weight (mg seedling^−1^); SDW—shoot dry weight (mg seedling^−1^); RDW—root dry weight (mg seedling^−1^); STWC—shoot tissue water content (mg H_2_O mg^−1^ shoot FW); RTWC—root tissue water content (mg H_2_O mg^−1^ root FW); RSR—root-shoot weight ratio; SVI—seedling vigor index; LRWC—leaf relative water content (%); CMS—cell membrane stability (%)).

**Table 3:** Extracted Eigenvalues and latent vectors of seedling traits associated with the first three principal components.

Variable	Principal Components		
	PC1	PC2	PC3
Extracted Eigenvalues	4.24	2.95	1.39
Explained variance (%)	35.4	24.6	11.6
Cumulative variance (%)	35.4	60.0	71.6
*Seedling traits*	*Latent vectors*		
Shoot length (SL)	0.793	−0.015	−0.147
Root length (RL)	0.642	0.111	0.113
Shoot fresh weight (SFW)	0.744	0.221	−0.359
Root fresh weight (RFW)	0.522	0.685	−0.071
Shoot dry weight (SDW)	0.606	−0.573	−0.217
Root dry weight (RDW)	0.436	0.482	0.590
Shoot tissue water content (STWC)	0.266	0.747	−0.223
Root tissue water content (RTWC)	0.370	0.514	−0.517
Root-shoot weight ratio (RSR)	−0.126	0.814	0.528
Seedling vigor index (SVI)	0.799	−0.214	0.093
Leaf relative water content (LRWC)	0.688	−0.396	0.347
Cell membrane stability (CMS)	0.696	−0.414	0.327

**Table 4:** Coefficients of linear discriminants of seedling traits associated with the three linear discriminant functions (LD) (traits ordered by the absolute size of the coefficients in LD1).

Variable	Linear Discriminants		
	LD1	LD2	LD3
Proportion of trace (%)	72.1	18.4	9.5
*Seedling traits*	*Coefficients*		
Root dry weight (RDW)	1.340	0.589	0.582
Shoot dry weight (SDW)	−0.729	−0.836	0.048
Shoot tissue water content (STWC)	0.640	−0.405	1.058
Cell membrane stability (CMS)	−0.538	0.294	−0.333
Root tissue water content (RTWC)	0.518	0.274	0.399
Leaf relative water content (LRWC)	−0.513	0.065	−0.438
Shoot length (SL)	−0.433	−0.406	−0.058
Seedling vigor index (SVI)	−0.316	−1.008	0.423
Root length (RL)	−0.255	−0.388	−0.304
Shoot fresh weight (SFW)	−0.165	0.346	−0.417
Root fresh weight (RFW)	0.134	−0.171	0.042
Root-shoot weight ratio (RSR)	−0.112	−1.238	−1.631

**Table 5:** Classification matrix of four clusters of 127 wheat genotypes according to linear discriminant analysis (rows being observed category and columns predicted category).

Clusters	True Groups				Total No. Observed
	Cluster 1	Cluster 2	Cluster 3	Cluster 4	
Cluster 1	18	0	3	1	22
Cluster 2	0	47	0	0	47
Cluster 3	1	8	24	0	33
Cluster 4	0	0	0	25	25
Total number	19	55	27	26	127
Number corrects	18	47	24	25	114
% correctness	94.7	85.5	88.9	96.2	89.8

**Table 6:** Pairwise Mahalanobis squared distances (*D*^2^) between four clusters of wheat genotypes after dimensionality reduction by LDA.

Clusters	Cluster 1	Cluster 2	Cluster 3	Cluster 4
Cluster 1	0	20.29 ^a^	11.51 ^a^	10.88 ^a^
Cluster 2	-	0	7.05 ^a^	21.02 ^a^
Cluster 3	-	-	0	17.23 ^a^
Cluster 4	-	-	-	0

^a^ Distances differing from zero at a 95% confidence interval.

**Table 7:** Best performers of 127 wheat genotypes within different co-cluster combinations under PEG-induced drought stress.

Co-Cluster Combinations		Best Performers
RC-1	CC-1	BD-684 followed by BD-526, BARI Gom 26, BARI Gom 29 and BARI Gom 24
	CC-2	BD-9913 followed by BD-588, BARI Gom 28, BD-526 and BARI Gom 19
	CC-3	BARI Gom 33 followed by BD-567, BD-580, BD-600 and BARI Gom 19
RC-2	CC-1	BD-553 followed by BD-9905, BD-623, BD-9915 and BD-9892
	CC-2	BD-637 followed by BD-9910, AS-10617, BD-4639 and BD-488
	CC-3	BD-9891 followed by BD-587, BD-9910, BD-639 and BD-477
RC-3	CC-1	BD-622 followed by BD-631, BD-9895, BD-9897 and BD-9896
	CC-2	AS-10638 followed by BD-7479, BD-622, BD-9114 and BD-466
	CC-3	BD-476 followed by BD-467, BD-550, BD-623 and BD-1271
RC-4	CC-1	BD-574 followed by BD-604, BD-9911, BD-525 and BARI Gom 20
	CC-2	BD-525 followed by BD-604, BARI Gom 30, BD-495 and BARI Gom 27
	CC-3	BD-666 followed by BD-494, BD-616, BD-523 and AS-10632

RC—row cluster; CC—column cluster; CC-1 (SDW, LRWC and CMS), CC-2 (RDW, STWC, RTWC and RSR) and CC-3 (SL, RL, SFW, RFW and SVI).

## Data Availability

The data that support the findings of this study are available from the corresponding authors upon reasonable request.

## Supplementary Materials

The following are available online at https://www.mdpi.com/article/10.3390/plants10050879/s1, Figure S1: Sample of experimental setup in the sand filled petriplates. Panel A showing condition of seedlings of a tolerant genotype BD-9889 (belongs to cluster 4) in both control and 25% PEG, and panel B showing the same of a sensitive genotype BD-623 (belongs to cluster 2), Figure S2: Gap statistic showing an optimal number of clusters to be created in the hierarchical cluster analysis based on STIs of the seedling traits, Table S1: List of wheat genotypes used in the exploratory study, Table S2: Mean squares and their effects on seedling traits extracted from the ANOVA of the general linear model, and Table S3: List of wheat genotypes of different clusters extracted by hierarchical co-clustering.

## Abbreviations

BBS	Bangladesh bureau of statistics
CRD	Completely randomized design
CMS	Cell membrane stability
EMS	Ethyl methanesulfonate
FAO	Food and agriculture organization
IQR	Interquartile range
LD	Linear discriminant
LDA	Linear discriminant analysis
LRWC	Leaf relative water content
MoEF	Ministry of environment and forest
PC	Principal component
PCA	Principal component analysis
PEG	Polyethylene glycol
PPED	Photoperiod with photosynthetic photon flux density
Q1	First quartile (25th Percentile)
Q3	Third quartile (75th Percentile)
RDW	Root dry weight
RFW	Root fresh weight
RH	Relative humidity
RHCOC	Robust hierarchical co-cluster
RL	Root length
RSR	Root-shoot weight ratio
RTWC	Root tissue water content
RWC	Relative water content
SDW	Shoot dry weight
SFW	Shoot fresh weight
SL	Shoot length
STI	Stress tolerance index
STWC	Shoot tissue water content
SVI	Seedling vigor index
