# Supplementary material for: Multivariate Analysis of Morpho-Physiological Traits Reveals Differential Drought Tolerance Potential of Bread Wheat Genotypes at the Seedling Stage
Source: Plants (Basel). 2021 Apr 27;10(5):879. doi: 10.3390/plants10050879 (PMC8145240; doi:10.3390/plants10050879)
Supplement: Supplementary file 1 [file plants-10-00879-s001.zip › plants-1169460-supplementary.pdf]

Table S1. List of wheat genotypes used in the exploratory study

Sl. No.	Genotype	Source	Type	*Germination %	
				Control	PEG
1	AS-10608	ACI Seed	Mutant	33.00	33.67
2	AS-10610	ACI Seed	Mutant	37.00	36.33
3	AS-10611	ACI Seed	Mutant	41.67	41.67
4	AS-10613	ACI Seed	Mutant	33.00	33.67
5	AS-10617	ACI Seed	Mutant	64.33	64.33
6	AS-10621	ACI Seed	Mutant	69.67	69.33
7	AS-10626	ACI Seed	Mutant	33.67	33.00
8	AS-10627	ACI Seed	Mutant	39.33	39.33
9	AS-10629	ACI Seed	Mutant	46.00	45.67
10	AS-10632	ACI Seed	Mutant	77.00	77.67
11	AS-10633	ACI Seed	Mutant	65.00	64.33
12	AS-10636	ACI Seed	Mutant	87.33	90.67
13	AS-10637	ACI Seed	Mutant	54.00	54.67
14	AS-10638	ACI Seed	Mutant	43.33	43.33
15	BARI Gom 19	BWMRI	Variety	74.67	74.00
16	BARI Gom 20	BWMRI	Variety	76.67	76.00
17	BARI Gom 21	BWMRI	Variety	53.67	54.00
18	BARI Gom 22	BWMRI	Variety	75.67	74.67
19	BARI Gom 23	BWMRI	Variety	77.33	75.67
20	BARI Gom 24	BWMRI	Variety	75.33	76.00
21	BARI Gom 25	BWMRI	Variety	68.00	67.33
22	BARI Gom 26	BWMRI	Variety	77.33	77.33
23	BARI Gom 27	BWMRI	Variety	82.00	82.00
24	BARI Gom 28	BWMRI	Variety	51.67	53.33
25	BARI Gom 29	BWMRI	Variety	74.00	72.67
26	BARI Gom 30	BWMRI	Variety	82.67	87.00
27	BARI Gom 31	BWMRI	Variety	81.33	82.00
28	BARI Gom 32	BWMRI	Variety	77.00	76.67
29	BARI Gom 33	BWMRI	Variety	66.33	64.67
30	BAW-1147	BWMRI	Advanced line	84.67	83.67
31	BD-1271	PGRC, BARI	Accession	34.33	34.00
32	BD-431	PGRC, BARI	Accession	44.67	44.33
33	BD-4633	PGRC, BARI	Accession	45.00	45.00

Sl. No.	Genotype	Source	Type	*Germination %	
				Control	PEG
34	BD-4638	PGRC, BARI	Accession	43.67	44.00
35	BD-4639	PGRC, BARI	Accession	54.00	53.67
36	BD-466	PGRC, BARI	Accession	66.00	65.00
37	BD-467	PGRC, BARI	Accession	78.00	74.00
38	BD-468	PGRC, BARI	Accession	62.67	64.67
39	BD-474	PGRC, BARI	Accession	80.67	80.00
40	BD-476	PGRC, BARI	Accession	62.00	63.00
41	BD-477	PGRC, BARI	Accession	90.67	89.67
42	BD-481	PGRC, BARI	Accession	59.00	57.00
43	BD-484	PGRC, BARI	Accession	62.33	61.00
44	BD-485	PGRC, BARI	Accession	65.67	66.00
45	BD-486	PGRC, BARI	Accession	55.00	54.00
46	BD-488	PGRC, BARI	Accession	65.67	64.33
47	BD-489	PGRC, BARI	Accession	61.67	62.00
48	BD-494	PGRC, BARI	Accession	71.67	71.67
49	BD-495	PGRC, BARI	Accession	71.67	70.33
50	BD-496	PGRC, BARI	Accession	48.33	47.00
51	BD-501	PGRC, BARI	Accession	51.67	52.00
52	BD-523	PGRC, BARI	Accession	65.67	67.33
53	BD-525	PGRC, BARI	Accession	73.33	73.00
54	BD-526	PGRC, BARI	Accession	64.67	65.00
55	BD-532	PGRC, BARI	Accession	61.67	62.00
56	BD-533	PGRC, BARI	Accession	46.67	46.33
57	BD-550	PGRC, BARI	Accession	66.33	66.67
58	BD-553	PGRC, BARI	Accession	33.33	34.00
59	BD-554	PGRC, BARI	Accession	55.67	55.67
60	BD-557	PGRC, BARI	Accession	72.33	71.67
61	BD-561	PGRC, BARI	Accession	46.33	45.67
62	BD-566	PGRC, BARI	Accession	52.67	53.00
63	BD-567	PGRC, BARI	Accession	62.33	62.33
64	BD-574	PGRC, BARI	Accession	70.00	69.67
65	BD-575	PGRC, BARI	Accession	65.33	64.33
66	BD-576	PGRC, BARI	Accession	66.00	65.67
67	BD-578	PGRC, BARI	Accession	54.00	53.00

Sl. No.	Genotype	Source	Type	*Germination %	
				Control	PEG
68	BD-580	PGRC, BARI	Accession	64.33	64.00
69	BD-583	PGRC, BARI	Accession	63.67	63.67
70	BD-585	PGRC, BARI	Accession	35.00	33.67
71	BD-587	PGRC, BARI	Accession	57.67	56.67
72	BD-588	PGRC, BARI	Accession	63.33	63.67
73	BD-591	PGRC, BARI	Accession	63.33	61.67
74	BD-592	PGRC, BARI	Accession	53.33	55.00
75	BD-594	PGRC, BARI	Accession	65.67	66.67
76	BD-597	PGRC, BARI	Accession	52.00	52.67
77	BD-600	PGRC, BARI	Accession	77.33	77.00
78	BD-604	PGRC, BARI	Accession	69.67	68.33
79	BD-605	PGRC, BARI	Accession	68.33	68.00
80	BD-616	PGRC, BARI	Accession	65.33	65.67
81	BD-617	PGRC, BARI	Accession	75.00	73.67
82	BD-618	PGRC, BARI	Accession	42.67	44.33
83	BD-622	PGRC, BARI	Accession	55.33	55.33
84	BD-623	PGRC, BARI	Accession	68.67	68.33
85	BD-631	PGRC, BARI	Accession	46.33	46.33
86	BD-637	PGRC, BARI	Accession	67.67	65.33
87	BD-639	PGRC, BARI	Accession	64.00	64.33
88	BD-641	PGRC, BARI	Accession	40.00	40.33
89	BD-666	PGRC, BARI	Accession	61.67	62.67
90	BD-668	PGRC, BARI	Accession	55.67	54.67
91	BD-674	PGRC, BARI	Accession	35.67	34.67
92	BD-675	PGRC, BARI	Accession	41.00	41.33
93	BD-679	PGRC, BARI	Accession	35.33	35.00
94	BD-683	PGRC, BARI	Accession	65.00	64.00
95	BD-684	PGRC, BARI	Accession	58.00	58.67
96	BD-684-1	PGRC, BARI	Accession	50.00	49.67
97	BD-7447	PGRC, BARI	Accession	37.00	36.67
98	BD-7449	PGRC, BARI	Accession	45.00	45.33
99	BD-7464	PGRC, BARI	Accession	64.67	65.00
100	BD-7479	PGRC, BARI	Accession	62.67	66.00
101	BD-8997	PGRC, BARI	Accession	35.33	34.33

Sl. No.	Genotype	Source	Type	*Germination %	
				Control	PEG
102	BD-9114	PGRC, BARI	Accession	39.00	38.67
103	BD-9186	PGRC, BARI	Accession	53.67	55.67
104	BD-9889	PGRC, BARI	Accession	75.33	75.67
105	BD-9891	PGRC, BARI	Accession	65.33	65.00
106	BD-9892	PGRC, BARI	Accession	62.00	62.67
107	BD-9895	PGRC, BARI	Accession	34.67	35.00
108	BD-9896	PGRC, BARI	Accession	32.00	32.00
109	BD-9897	PGRC, BARI	Accession	41.00	42.33
110	BD-9898	PGRC, BARI	Accession	34.67	32.67
111	BD-9900	PGRC, BARI	Accession	55.33	55.00
112	BD-9901	PGRC, BARI	Accession	65.33	64.33
113	BD-9905	PGRC, BARI	Accession	64.67	65.00
114	BD-9906	PGRC, BARI	Accession	66.33	66.00
115	BD-9908	PGRC, BARI	Accession	45.33	46.67
116	BD-9910	PGRC, BARI	Accession	85.00	86.33
117	BD-9911	PGRC, BARI	Accession	75.33	75.00
118	BD-9913	PGRC, BARI	Accession	66.33	65.00
119	BD-9914	PGRC, BARI	Accession	66.00	66.00
120	BD-9915	PGRC, BARI	Accession	54.67	56.00
121	BD-9918	PGRC, BARI	Accession	88.33	88.33
122	BD-9919	PGRC, BARI	Accession	43.00	44.00
123	BD-9922	PGRC, BARI	Accession	33.67	33.33
124	BD-9930	PGRC, BARI	Accession	71.33	74.00
125	BD-9997	PGRC, BARI	Accession	64.67	64.33
126	BINA wheat 1	BINA	Variety	67.33	65.33
127	Kanchan	BWMRI	Variety	81.33	81.33

*Germination percent was calculated before treatment imposition, i.e., wheat seeds were allowed to germinate in the same setup and then PEG-6000 was applied to one set.

ACI- Advanced Chemical Industries; BARI- Bangladesh Agricultural Research Institute; BINA-Bangladesh Institute of Nuclear Agriculture; BWMRI- Bangladesh Wheat and Maize Research Institute; PGRC- Plant Genetic Resource Center.

Table S2. Mean squares and their effects on seedling traits extracted from the ANOVA of the general linear model

Source of variation	DF	Mean squares											
		SL	RL	SFW	RFW	SDW	RDW	STWC	RTWC	RSR	SVI	LRWC	CMS
Genotype (G)	126	32.2**	24.2**	1130**	562.6**	18.2**	7.2**	0.015**	0.022**	0.187**	102.9**	300.7**	244**
Treatment (T)	1	2860.4**	1244.7**	133953**	22853**	815.1**	165.4**	0.217**	0.325**	0.826**	2351**	28875**	50066**
G×T	126	7.5**	5.8**	189**	51.3**	1.3**	0.4**	0.0004	0.0012**	0.008**	5.9**	6.2**	3.0
Residual	508	0.7	0.4	10	2.9	0.4	0.2	0.0006	0.0006	0.0005	0.15	3.9	3.0

** and * indicate significant at $P \leq 0.01$ and $P \leq 0.05$, respectively.

Table S3. List of wheat genotypes of different clusters extracted by hierarchical co-clustering

Row clusters	No. of genotypes	Name of the genotypes
Cluster 1	19	BARI Gom 19, BARI Gom 24, BARI Gom 26, BARI Gom 28, BARI Gom 29, BARI Gom 33, BD-485, BD-526, BD-554, BD-557, BD-567, BD-580, BD-588, BD-600, BD-684, BD-9913, BD-9919, BD-9922, and BD-9930.
Cluster 2	55	AS-10608, AS-10610, AS-10611, AS-10613, AS-10617, AS-10621, AS-10626, AS-10627, AS-10629, AS-10633, AS-10636, BARI Gom 21, BD-4639, BD-468, BD-474, BD-477, BD-486, BD-488, BD-489, BD-496, BD-501, BD-532, BD-533, BD-553, BD-561, BD-566, BD-578, BD-583, BD-587, BD-591, BD-592, BD-617, BD-623, BD-639, BD-641, BD-668, BD-674, BD-675, BD-679, BD-683, BD-684-1, BD-7464, BD-8997, BD-9186, BD-9891, BD-9892, BD-9900, BD-9901, BD-9905, BD-9908, BD-9910, BD-9914, BD-9915, BD-9997, and BINA wheat 1.
Cluster 3	27	AS-10637, AS-10638, BD-1271, BD-431, BD-4633, BD-4638, BD-466, BD-467, BD-476, BD-484, BD-550, BD-585, BD-597, BD-605, BD-618, BD-622, BD-631, BD-637, BD-7447, BD-7449, BD-7479, BD-9114, BD-9895, BD-9896, BD-9897, BD-9898, and BD-9918.
Cluster 4	26	AS-10632, BARI Gom 20, BARI Gom 22, BARI Gom 23, BARI Gom 25, BARI Gom 27, BARI Gom 30, BARI Gom 31, BARI Gom 32, BAW-1147, BD-481, BD-494, BD-495, BD-523, BD-525, BD-574, BD-575, BD-576, BD-594, BD-604, BD-616, BD-666, BD-9889, BD-9906, BD-9911, and Kanchan.

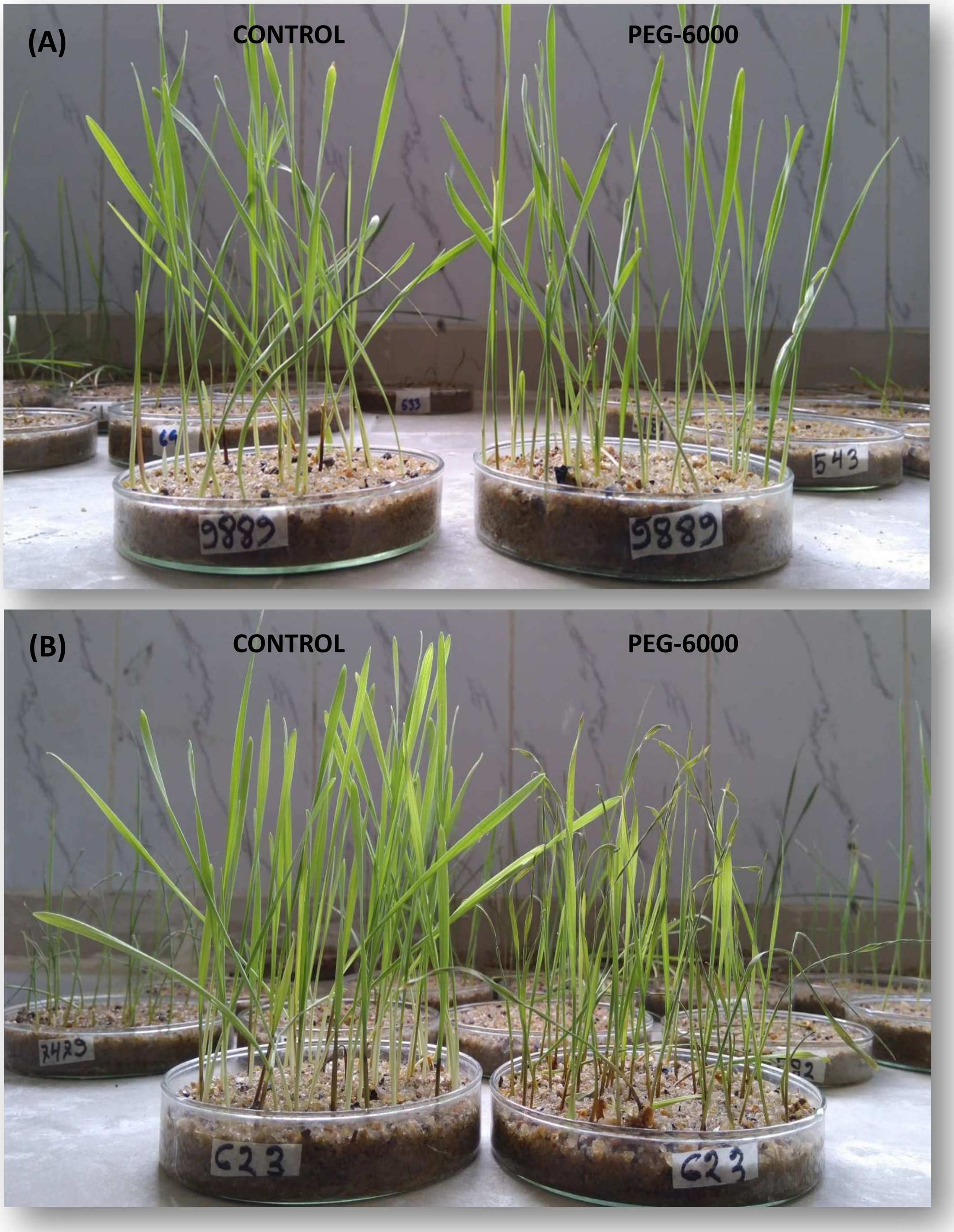

Figure S1. Sample of experimental setup in the sand filled petriplates. Panel (A) showing condition of seedlings of a tolerant genotype BD-9889 (belongs to cluster 4) in both control and 25% PEG, and panel (B) showing the same of a sensitive genotype BD-623 (belongs to cluster 2).

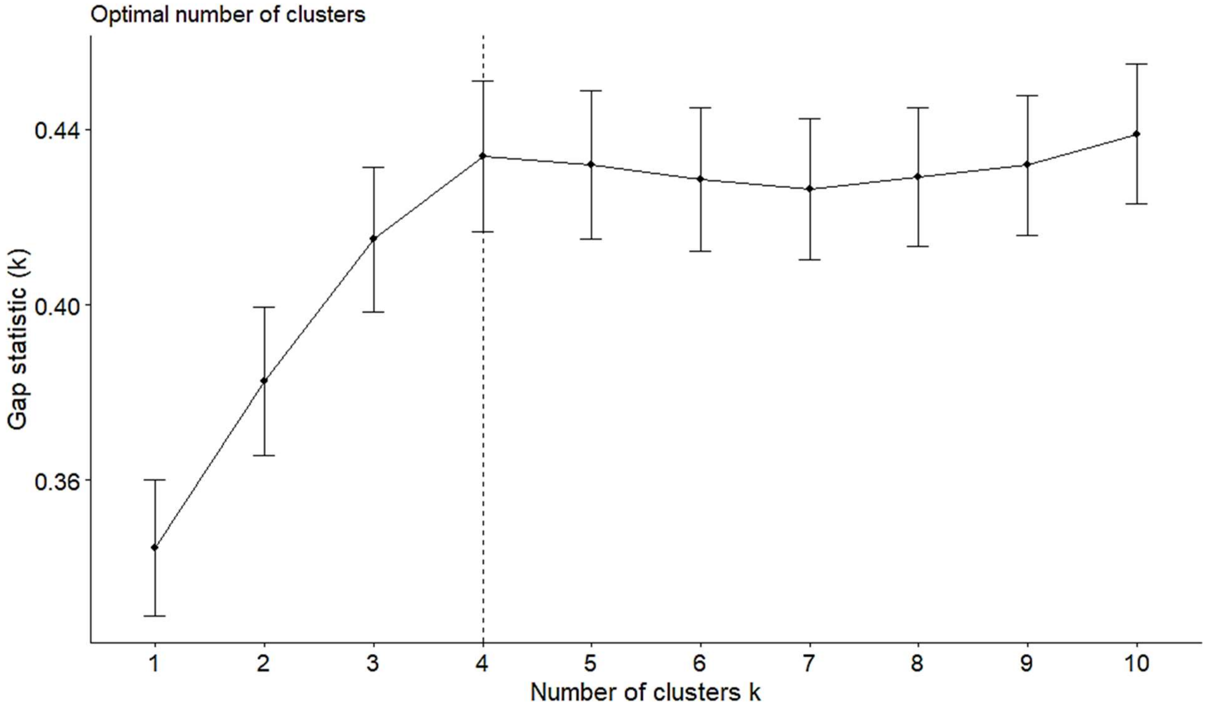

Figure S2. Gap statistic showing optimal number of clusters to be created in the hierarchical cluster analysis on the based on STIs of the seedling traits.
